# Dimethyl Fumarate-Induced Takotsubo Cardiomyopathy in a Patient With Relapsing-Remitting Multiple Sclerosis

**DOI:** 10.7759/cureus.23789

**Published:** 2022-04-03

**Authors:** Bahadar S Srichawla

**Affiliations:** 1 Neurology, University of Massachusetts Chan Medical School, Worcester, USA

**Keywords:** takotsubo cardiomyopathy, adult neurology, transthoracic echocardiogram, pulmonary and critical care medicine, neurology and critical care, disease modifying anti rheumatic drugs, dimethyl fumarate, cardiology, multiple sclerosis and other demyelinating disorders, neuro-immunology

## Abstract

Dimethyl fumarate (DMF) is an approved oral pharmacologic agent used in the treatment of relapsing-remitting multiple sclerosis (RRMS). Although commonly used in clinical practice, its mechanism of action remains largely unknown. Some frequent side effects associated with this drug are angioedema, hepatic injury, flushing, herpes zoster infection, and abdominal pain among others. A 47-year-old female presented with symptoms of an allergic reaction after initiating DMF therapy. She required intensive care unit admission due to an acute-hypoxic respiratory failure. A transthoracic echocardiogram (TTE) revealed apical ballooning and a left ventricular ejection fraction (LVEF) of 35%-40%. A coronary angiogram revealed no coronary artery disease. The diagnosis of takotsubo cardiomyopathy was made. The patient was managed with high-dose steroids and an epinephrine drip, in addition to a high-flow nasal cannula (HFNC) for respiratory support. At a three-month follow-up, a repeat TTE showed a resolution of the underlying takotsubo cardiomyopathy (CM) with no stunted myocardium and a normal ejection fraction (EF). Here, I highlight a life-threatening case of DMF-induced takotsubo CM and familiarize clinicians and patients with the need for close monitoring of symptoms when initiating disease-modifying drug (DMD) therapy.

## Introduction

Since 2013, dimethyl fumarate (DMF) has been approved by the U.S. Food and Drug Administration (FDA) for the treatment of relapsing-remitting multiple sclerosis (RRMS). RRMS is the most common form of multiple sclerosis (MS), and as its name suggests, it occurs due to the discrete onset of symptoms and a period of remission. Approximately 85% of patients diagnosed with MS have the relapsing-remitting variant, and over half of the patients diagnosed with MS have received disease-modifying drug (DMD) therapy [1]. DMF is a disease-modifying drug and is prescribed to be taken orally twice a day and is understood to have an acceptable tolerability profile [2].

Although the exact mechanism of action is unknown in RRMS, DMF is a known upregulator of the nuclear factor-erythroid (NF-ER)-related factor 2 (Nfr2) transcriptional pathway which leads to an increase in both synthesis and cellular recycling of glutathione. Glutathione is a known reactive oxygen species (ROS) scavenger in the central nervous system (CNS) [2]. The most common adverse events related to DMF therapy are skin flushing and gastrointestinal symptoms. Most adverse events (AEs) have been recorded to be mild to moderate and spontaneously resolved with continuous administration of DMF [3]. No known cases of DMF-induced takotsubo cardiomyopathy have been recorded in the scientific literature.

Takotsubo cardiomyopathy (TCM) is a reversible cardiomyopathy that often leads to dyskinesis of the left ventricle and apical ballooning. It is believed that catecholamines play a significant role in the pathogenesis of takotsubo cardiomyopathy, which is why it is often referred to as stress-induced cardiomyopathy [4]. Here, I am presenting a unique case of takotsubo cardiomyopathy after taking oral DMF which required ICU level of medical care. This case report aims to further our understanding of the AEs associated with DMF.

## Case presentation

**Figure 1:**
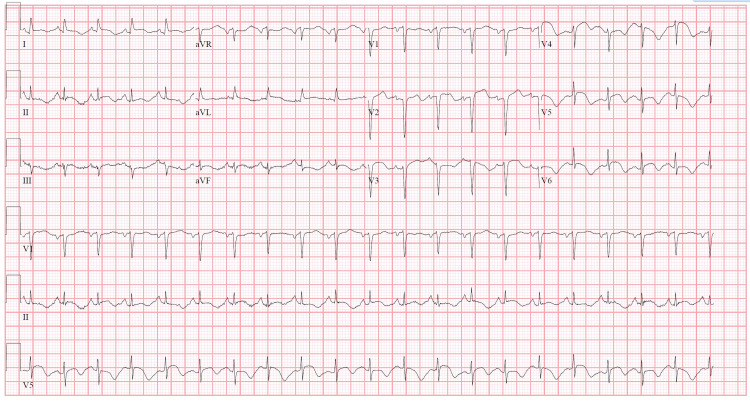
An electrocardiogram (ECG) showing sinus tachycardia and T-wave inversion in precordial leads/V3-V6. aVR: augmented vector right, aVL: augmented vector left, aVF: augmented vector foot.

A 47-year-old female with a known history of relapsing-remitting multiple sclerosis (RRMS) presented with subacute onset worsening chest pain, shortness of breath, skin flushing, and lower extremity edema after taking prescribed dimethyl fumarate (DMF) orally (120 mg twice a day) for the past three days. She stated that after taking the medication on the day of presentation, her dyspnea persisted at rest and she developed intermittent left-sided chest pain, diffuse skin flushing, and noted her legs looked swollen. It is understood that the patient had an allergic reaction to DMF and had no known allergies. Vital signs included blood pressure (BP) of 122/85 mmHg, heart rate of 142 beats per minute, respiratory rate of 33 breaths per minute, afebrile, and oxygen saturation of 86% on room air. Physical examination revealed erythema of the skin, most pronounced over the face and anterior chest wall, bilateral crackles with wheezing, raised jugular venous pressure (JVP), and 2+ pitting edema of the bilateral lower extremities to the midcalf. Neurologic examination was significant for 3+ hyperreflexia of the right patella. The patient was diagnosed with RRMS approximately eight years ago and has had relapsing episodes of leg stiffness. These recurrent episodes have progressively made it more difficult for her to walk. A venous blood gas (VBG) was obtained revealing pH 7.11, partial pressure of carbon dioxide (pCO_2_) 87.8 mmHg, partial pressure of oxygen (pO_2_) 38 mmHg, saturated O_2_ 51%, and HCO_3_ 27.5 mmol/L. ECG revealed sinus tachycardia and T-wave inversion in precordial leads (Figure 1).

A comprehensive metabolic profile (CMP), complete blood count (CBC), troponin, brain natriuretic peptide (BNP), and lactic acid were obtained as shown in Table 1. Notable values included leukocytosis, WBC, of 19,900 with an absolute neutrophil count (ANC) of 13,860.

**Table 1:** Complete blood count (CBC) with differential, comprehensive metabolic profile (CMP), non-high-sensitivity troponin, lactic acid, and brain natriuretic peptide (BNP) on presentation. BUN: blood urea nitrogen, eGFR: effective glomerular filtration rate, BSA: body surface area, AST: aspartate aminotransferase, ALT: alanine aminotransferase, MCV: mean corpuscular volume, MCH: mean corpuscular hemoglobin, MCHC: mean corpuscular hemoglobin concentration, RDW: red cell distribution width.

Lab parameter	Numerical value and normal range with units
Sodium	141 mmol/L (135-145 mmol/L)
Potassium	3.8 mmol/L (3.5-5.3 mmol/L)
Chloride	101 mmol/L (97-110 mmol/L)
CO_2_	23 mmol/L (24-32 mmol/L)
Anion gap	13
Glucose	240 mg/dL (70-99 mg/dL)
BUN	7 mg/dL (7-23 mg/dL)
Creatinine	0.80 mg/dL (0.60-1.30 mg/dL)
eGFR	>90 mL/min/BSA
Calcium	9.3 mg/dL (8.7-10.7mg/dL)
Phosphorus	5.8 mg/dL (2.5-4.5 mg/dL)
Total Protein	5.8 g/dL (6.0-8.0 g/dL)
Albumin	3.3 g/dL (3.5-4.8 g/dL)
Bilirubin, total	0.5 mg/dL (0.3-1.2 mg/dL)
Bilirubin, direct	0.1 mg/dL (<0.4 mg/dL)
Alkaline phosphatase	72 U/L (30-115 U/L)
AST	133 U/L (10-40 U/L)
ALT	302 U/L (10-40 U/L)
Lactic acid	2.8 mmol/L (0.1-1.6 mmol/L)
Troponin	2.04 ng/mL (<0.04 ng/mL)
Brain natriuretic peptide (BNP)	1268 pg/mL (<100 pg/mL)
WBC	19.9×10^3^ μL (4.3-10.8×10^3^ μL)
RBC	4.92×10^6^ μL (4.2-5.8×10^6^ μL)
Hemoglobin	14.9 g/dL (13.2-17.1 g/dL)
Hematocrit	44.8% (39.0-52.0 %)
MCV	91.1 fL (80.0-100.01 fL)
MCH	30.3 pg (27.0-34.0 pg )
MCHC	33.3 g/dL (29.0-36.0 g/dL)
RDW	13.1% (11.0%-15.0%)
Platelet count	361×10^3^ μL (140-44×10^3^ μL)
MPV	9.7 fL (7.6-11.6 fL)
Neutrophil %	69.7%
Lymphocyte %	11.3%
Monocyte %	5.4%
Eosinophil %	13.1%
Basophil %	0.6%
Absolute neutrophils	13.86×10^3^ μL (1.60-7.50×10^3^ μL)
Absolute lymphocytes	2.2×10^3^ μL (0.9-3.4×10^3^ μL)
Absolute monocytes	1.1×10^3^ μL (0.0-1.2×10^3^ μL)
Absolute eosinophils	2.6×10^3^ μL (0.0-0.6×10^3^ μL)
Absolute basophils	0.1×10^3^ μL (0.0-0.3×10^3^ μL)

The patient was double-vaccinated against COVID-19 with the mRNA-1273 vaccine approximately four months prior to presentation. Further microbiologic testing with a urine analysis (UA), two sets of blood cultures, urine cultures, respiratory culture with gram stain, Legionella urine antigen, severe acute respiratory syndrome coronavirus 2 (SARS-CoV-2) RNA polymerase chain reaction (PCR), and methicillin-resistant *Staphylococcus aureus* (MRSA) nasal swab were completed which later resulted in no growth. One month prior to presentation, the patient was found to have a negative QuantiFERON-Tuberculosis Gold Plus (Quest Diagnostics, Secaucus, United States) test and was non-reactive to the hepatitis A IgM antibody, hepatitis B core IgM antibody, hepatitis B surface antigen, and hepatitis C antibody.

A chest x-ray (CXR) revealed non-specific mild reticular markings of the bilateral lung bases. A computerized tomography (CT) scan of the chest with pulmonary embolus (PE) protocol was completed showing no PE. The patient was immediately given 0.3 mg of epinephrine and started on an epinephrine drip. Intravenous (IV) Solu-Medrol 125 mg was administered, and the patient was placed on bilevel positive airway pressure (BiPAP) with albuterol nebulizers. However, the patient did not tolerate BiPAP due to discomfort and was placed on a high-flow nasal cannula (HFNC) with a fraction of inspired oxygen (FiO_2_) of 60% at 60 L per minute and was transferred to the ICU for monitoring. On hospital day two, the troponin peaked at 4.47 and lactate at 5.50 with de-escalating oxygen requirements and the patient was transitioned to oral prednisone 60 mg daily. Cardiology was consulted, and a bedside TTE showed moderately reduced systolic function with a left ventricular ejection fraction of 35%-40% and akinesis of the LV apex suggestive of stress-induced cardiomyopathy (Video 1).

**Video 1:** Transthoracic echocardiogram (TTE) imaging showing a reduced systolic function of the left ventricle (LV) with an ejection fraction (EF) of 35%-40% and notable apical kinesis.

The patient was weaned to nasal cannula and was transferred to the general medicine wards for further management. On the subsequent day, the patient was on room air and underwent myocardial perfusion stress imaging which revealed no ischemic ECG changes. A coronary angiogram revealed no significant coronary artery disease (CAD). A myocardial biopsy was offered for further diagnostic testing; however, the patient declined. The patient was discharged on hospital day eight with a tapering dose of prednisone. At a three-month follow-up appointment, a repeat TTE was completed showing normal systolic function with an estimated LVEF of 60% and no apical ballooning (Video 2). The patient was subsequently started on ocrelizumab for the management of RRMS. At a six-month follow-up, the patient was tolerating the medication well and had not reported any adverse events.

**Video 2:** Repeat transthoracic echocardiogram (TTE) imaging three months after presentation revealing normal systolic function of the left ventricle (LV) and resolution of apical kinesis. EF: ejection fraction.

## Discussion

The most prevalent form of MS is the relapsing-remitting variant [5]. Traditionally, RRMS is treated with injectable immunomodulators; however, within the past decade, oral disease-modifying drugs (DMDs) such as dimethyl fumarate (DMF) have now begun to be used for management. Current studies have shown that DMF is believed to have a reassuring side effect profile [2,6]. However, this case describes a severe adverse effect to DMF. Based on the patient’s hospital course, it is reasonable to postulate takotsubo cardiomyopathy (TCM) occurred in an iatrogenic manner secondary to both intramuscular epinephrine administration and the initiation of an epinephrine drip. Not surprisingly, researchers in the past have attempted to delineate when TCM occurs as a response to an allergic reaction versus as a response to epinephrine [7-8]. However, the patient’s initial presentation of elevated JVP, pitting lower extremity edema, and crackles on physical exam prior to the administration of epinephrine does favor a drug-induced TCM rather than epinephrine. Stress-induced cardiomyopathies are also associated with elevation of troponin I (TnI) which was markedly elevated in our case and was obtained via point-of-care venous sampling prior to epinephrine administration [9].

DMF is known to cause mild-to-moderate adverse events, often gastrointestinal symptoms; however, most severe AEs have been reported to be secondary to immunocompromised status as seen in other DMDs [10]. Exact statistics on adverse events related to DMF are not known; however, most symptoms include gastrointestinal disturbances such as nausea, vomiting, and diarrhea. Some of the most severe reported AEs related to DMF therapy include severe lymphopenia and meningitis [11-12]. However, there are no reported cases of takotsubo cardiomyopathy secondary to disease-modifying drug therapy in the scientific literature. A proposed mechanism by which DMF exerts its anti-inflammatory effects is via the inactivation of glycolytic enzyme glyceraldehyde 3-phosphate dehydrogenase (GAPDH) which halts aerobic respiration in myeloid and lymphoid cells [13].

Catecholamines play a significant role in the pathogenesis of takotsubo cardiomyopathy. High levels of catecholamines binding to beta-2-adrenergic receptors lead to uncoupling of G-proteins and increased activation of the G_i_ protein exerting a negative ionotropic effect. The highest level of these beta-2 adrenergic receptors is found in the apex of the myocardium where apical ballooning is seen in stress cardiomyopathy [14]. Cessation of the acute stressor leads to normalization of catecholamine levels which allows the cardiomyopathy to be reversed. Thus, medications that lead to increased circulating catecholamines are implicated in the pathogenesis of takotsubo cardiomyopathy. Some common offenders include epinephrine, ephedrine, levothyroxine, and venlafaxine. Beta-blocker and oxycodone withdrawal are also implicated [14].

Increased concentrations of reactive oxygen species (ROS) are known to increase levels of circulating catecholamines. In one of the in vitro studies, DMF has been shown to transiently lower glutathione (GSH) levels, a known scavenger of ROS [15]. One hypothesis is that DMF led to decreased levels of GSH, and a reflexive rise in catecholamine levels played a role in the pathogenesis of takotsubo cardiomyopathy. Larger studies looking at the temporal relationship of DMF therapy dependent on a decrease in GSH are needed. In our case, takotsubo cardiomyopathy may have occurred directly due to DMF therapy or indirectly as the result of catecholamine release from an allergic reaction to DMF.

Few cases of takotsubo cardiomyopathy (TCM) in patients with multiple sclerosis (MS) have been recorded. One patient on DMF therapy for MS developed takotsubo cardiomyopathy 10 days after developing symptoms of COVID-19 [16]. TCM in this case is believed to be due to infection with SARS-CoV-2 and not due to DMF. An MS exacerbation with lesions to the vasomotor center or nucleus of the solitary tract may lead to increased sympathetic output precipitating a catecholamine surge [16]. A similar case of TCM has been reported as an initial clinical presentation for MS [17]. In this case, the patient was managed with a three-day course of methylprednisolone with a favorable response. In our patient, the initial presentation of MS and future exacerbations was lower extremity weakness and no episodes of TCM or cardiac disease were present. The Naranjo Adverse Drug Reaction Scale (Naranjo scale) is used to determine whether the therapeutic dosing of medication caused an adverse event or not. As shown in Table 2, a total of five points are scored [18]. Based on the Naranjo scale, TCM is a probable adverse event from DMF administration in our case.

**Table 2:** Naranjo Adverse Drug Reaction (ADR) Scale. 0: doubtful ADR; 1-4: possible ADR; 5-8: probable ADR; >9: definite ADR.

Question	Yes	No	Do Not Know
Are there previous conclusive reports on this reaction?	+1	0	0
Did the adverse event appear after the suspected drug was administered?	+2	-1	0
Did the adverse event improve when the drug was discontinued or a specific antagonist was administered?	+1	0	0
Did the adverse event reappear after the drug was readministered?	+2	-1	0
Are there alternative causes that could on their own have caused this reaction?	-1	+2	0
Did the reaction reappear when a placebo was given?	-1	+1	0
Was the drug detected in blood or other fluids in concentrations known to be toxic?	+1	0	0
Was the reaction more severe when the dose was increased or less severe when the dose was decreased?	+1	0	0
Did the patient have a similar reaction to the same or similar drugs in any previous exposure?	+1	0	0
Was the adverse event confirmed by any objective evidence?	+1	0	0

Further research is still warranted on the exact mechanism of action of DMF therapy in RRMS, and given its immunomodulatory effects, its safety profile must continue to be monitored. However, given the life-threatening reaction seen here and its known effects on the immune system, it is recommended to closely monitor symptoms after beginning DMF therapy. If a severe AE is noted, DMF therapy should be discontinued altogether.

## Conclusions

Dimethyl fumarate (DMF) is an FDA-approved oral disease-modifying drug (DMD) used in the treatment of relapsing-remitting multiple sclerosis. It is a known immunomodulator; however, the exact mechanism of action remains unknown. DMF is known to generally have a reassuring safety profile; however, this case highlights that DMDs such as dimethyl fumarate can cause severe and rare adverse events (AEs) such as stress-induced (takotsubo) cardiomyopathy. It is recommended to closely monitor for symptoms after beginning any DMD and to cease therapy henceforth if a severe AE is measured. Further studies on the exact mechanism of action of DMF and its immunomodulatory effects are needed.
